# Achieving a Mode-Selective Optical Waveguide in a PIN-PMN-PT Single Crystal via a Nickel In-Diffusion Method

**DOI:** 10.3390/nano16090514

**Published:** 2026-04-24

**Authors:** Yuebin Zhang, Qingyuan Hu, Xin Liu, Yongyong Zhuang, Binbin Zhang, Wentao Yang, Lunan Gao, Zhe Liu, Yifan Zhang, Wenxu Huang, Yali Feng, Lei An, Zhuo Xu, Xiaoyong Wei

**Affiliations:** Electronic Materials Research Laboratory, Key Laboratory of the Ministry of Education & International Center for Dielectric Research, School of Electronic Information, Xi’an Jiaotong University, Xi’an 710049, China; zybzgxx@163.com (Y.Z.); eudoraliu@xjtu.edu.cn (X.L.); zhuangzhuang235@xjtu.edu.cn (Y.Z.); zbb001@stu.xjtu.edu.cn (B.Z.); 4125153046@stu.xjtu.edu.cn (W.Y.); gaolunan@stu.xjtu.edu.cn (L.G.); 2733563282@stu.xjtu.edu.cn (Z.L.); zpang2022@163.com (Y.Z.); wenxu200031@163.com (W.H.); fengyalifly@163.com (Y.F.); anlei0122@163.com (L.A.); xuzhuo@mail.xjtu.edu.cn (Z.X.)

**Keywords:** ferroelectric crystal, PIN-PMN-PT, nickel in-diffusion, waveguide

## Abstract

Relaxor ferroelectric single crystals, such as Pb(In_1_/_2_Nb_2_/_3_)O_3_–Pb(Mg_1_/_2_Nb_2_/_3_)O_3_–PbTiO_3_, possess extraordinary electro-optic (EO) coefficients, offering immense potential for next-generation integrated modulators. However, the application of PIN-PMN-PT in fiber-optic gyroscopes (FOGs) is hindered by the challenge of fabricating high-quality optical waveguides with strict mode selectivity, as conventional diffusion typically excites multi-mode propagation. Here, the fabrication of high-quality, mode-selective waveguides is achieved in rhombohedral PIN-PMN-PT via a nickel in-diffusion technique. The resulting graded-index structures exhibit a Gaussian profile with a maximum refractive index change (∆*n*) of 1.53% while preserving the single crystal structure. Under specific processing conditions, we achieve precise mode selectivity, enabling exclusive transverse electric (TE) mode transmission. This mode selectivity fulfills the requirements for single-mode Y-branch geometries, establishing a robust platform for ultra-compact, low driving voltage modulators and advancing the miniaturization of inertial navigation and integrated photonic systems.

## 1. Introduction

Electro-optic modulators, which convert electrical signals into optical signals, are critical components in optical communications, microwave photonics, and inertial navigation systems [1,2,3,4]. However, the performance requirements for electro-optic modulators vary significantly across different applications. For high-speed optical communication and microwave photonic systems, modulators require high integration density and broad modulation bandwidth [5,6]. In contrast, for fiber optic gyroscopes (FOGs)—essential sensing elements in inertial navigation systems—the emphasis shifts toward miniaturization and low power consumption. This focus is driven by the ongoing demand for next-generation FOGs with higher accuracy, reduced size, and improved energy efficiency [7,8]. The performance of an electro-optic modulator is largely determined by its constituent materials. Among various candidate materials, lithium niobate (LN) crystals are widely used due to their strong electro-optic coefficient, low optical loss, and broad transparency window [9,10,11,12]. For example, Y-branch waveguide modulators based on LN are core components in fiber optic gyroscopes. However, the electro-optic coefficient of conventional LN single crystals (typically 20–30 pm/V) has reached a performance plateau, limiting the further improvement of key device parameters such as driving voltage and footprint [13,14,15].

The principle of modulators based on electro-optic crystals can be described by the following equation [16]:

(1) $$V_{\pi }\cdot L=\frac{\lambda g}{n_{o}^{3}\gamma_{33}\Gamma }$$

The performance of an electro-optic modulator is critically determined by the product of its half-wave voltage (*V_π_*) and length (*L*), which is inversely proportional to the electro-optic coefficient (*γ*_33_). Therefore, a higher electro-optic coefficient is essential for developing compact, low-power-consumption integrated modulators [17]. Recently, a remarkable electro-optic coefficient of up to 900 pm/V—an order of magnitude larger than that of lithium niobate—has been achieved in transparent PIN-PMN-PT crystals via a high-temperature poling technique [18,19]. While its exceptional properties position PIN-PMN-PT as an ideal platform for integrated photonics, realizing this potential requires high-quality optical waveguides. Previous efforts to fabricate waveguides in PIN-PMN-PT have been reported. Hu et al. demonstrated a titanium-diffused planar waveguide in PIN-PMN-PT, achieving a refractive index change of approximately 3% and subsequently, a modulator with a *V_π_*·*L* product of 1.38 V·cm [20,21]. Specific applications, such as fiber-optic gyroscopes, require electro-optic modulators with Y-branch geometries and strict mode selectivity [22,23]. However, titanium-diffused PIN-PMN-PT waveguides typically support multiple guided modes, precluding the single-mode operation necessary for these devices [24]. Consequently, alternative diffusion elements with different characteristics must be explored to create mode-selective waveguides. Nickel (Ni) is one such candidate, offering advantages such as a lower diffusion temperature and the absence of an out-diffusion layer. Furthermore, the guiding properties of Ni-diffused waveguides can be tailored by adjusting diffusion parameters for different applications [25,26]. Schmidt et al. demonstrated that this technique can be tailored to support exclusively TE mode in LiNbO_3_ single crystals under specific diffusion conditions [27]. Consistent with this, Chen et al. reported that ridge waveguides defined by 5 μm wide nickel stripes successfully guided a single mode, either TE or transverse magnetic (TM), at a wavelength of 633 nm, confirming the potential for precise modal control [28]. These findings suggest that nickel diffusion is a promising method for achieving mode selectivity in PIN-PMN-PT optical waveguides.

In this work, we report the fabrication of a nickel-diffused waveguide in a rhombohedral 0.21PIN-0.51PMN-0.28PT single crystal. We systematically investigated the effects of nickel diffusion on the crystal structure using scanning electron microscopy (SEM) and X-ray diffraction (XRD). The waveguide modes and the refractive index profile were characterized using a prism coupler. The resulting waveguide exhibits a high refractive index contrast, excellent mode selectivity, and low optical propagation loss.

## 2. Experimental

### 2.1. Preparation of the Highly Transparent PIN-PMN-PT Crystal

The single crystal used in this study was a ternary relaxor ferroelectric PIN-PMN-PT, grown via the modified Bridgman method [29]. This ternary system maintains piezoelectric and electro-optic properties comparable to binary PMN-PT crystals but exhibits a significantly higher Curie temperature which greatly enhances its thermal stability for applications requiring a wider operational temperature range [30]. The key performance metrics of PIN-PMN-PT crystals, including optical transmittance, refractive index, and electro-optic coefficients, are strongly influenced by crystal orientation and composition. For instance, rhombohedral-phase PIN-PMN-PT can achieve a transmittance of up to 65% in the as-grown state, which increases to approximately 71% after poling—a value nearing the theoretical limit when interfacial reflection is accounted for [31]. The refractive index, which is consistently higher than that of lithium niobate (LiNbO_3_), gradually increases with rising PT content. Notably, through synergistic optimization of composition, orientation, and domain structure, an exceptional electro-optic coefficient of nearly 900 pm/V has been achieved in [011]-poled rhombohedral PIN-PMN-PT crystals [19]. For growing large-size crystals, the two primary methodologies are growth along the [001] or [011] directions. Achieving high refractive index uniformity for light propagation along the [001] optical axis demands exceptional compositional homogeneity along that direction. Therefore, this work utilized PIN-PMN-PT crystals grown along the [011] direction. Wafers were sliced perpendicular to the growth axis, the [001] direction within the wafer was identified, and samples were subsequently cut for experimentation.

### 2.2. Nickel In-Diffusion Processing

The PIN-PMN-PT crystal was thoroughly cleaned before processing. A 50 nm-thick nickel (Ni) film was then deposited on the crystal surface using RF magnetron sputtering. Subsequent thermal diffusion was carried out in a tube furnace at 800–900 °C for 6–10 h. To ensure uniform diffusion and minimize thermal stress, a controlled cooling procedure was applied: the temperature was first lowered to 650 °C at 1 °C/min and held for 2 h, then further cooled to 250 °C, followed by a slow cooling rate of 6 °C/h down to 150 °C, before finally cooling naturally to room temperature. To prevent lead loss from the crystal at high temperatures, the diffusion process was conducted in a lead-rich atmosphere. This was achieved by placing the sample in a sealed alumina crucible embedded with lead oxide (PbO) powder. Upon heating, the PbO powder vaporized, creating the required Pb-rich environment around the crystal.

### 2.3. Characterization of the PIN-PMN-PT Waveguide

A dense nickel film was sputter-deposited using a high-purity (99.995%) target, and the un-diffused sample surfaces were precision-ground to the target thickness. The structural and elemental properties of the nickel-diffused waveguide layer were systematically characterized. Crystal phase structure was analyzed via X-ray diffraction (XRD, D/Max-IIIC, Rigaku, Japan) using Cu Kα radiation, while elemental concentration profiles and diffusion depths were determined via X-ray photoelectron spectroscopy (XPS) and energy-dispersive X-ray spectroscopy (EDS), respectively. For cross-sectional scanning electron microscopy (SEM, Carl Zeiss Merlin, Germany), the samples were meticulously ground, polished to prevent surface damage, and gold-coated. Optically, crystal transmittance and the effective electro-optic coefficient were determined using a spectrophotometer (Jasco V570, Japan) and a custom-built single-beam ellipsometer, respectively. Waveguide modes were characterized at 633 nm using a prism coupler (Model 2010/M, USA) equipped with a symmetric GaP prism. Finally, we evaluated the dependence of the modal refractive index on diffusion times (6 h and 10 h) at fixed temperatures of 800 °C and 900 °C.

## 3. Results and Discussion

### 3.1. Electro-Optic Property of the PIN-PMN-PT Crystal

**Figure 1:**
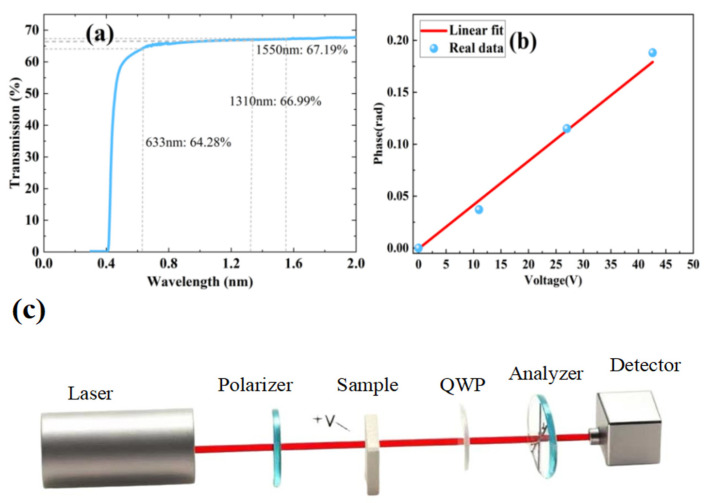
(**a**) Transmission spectrum of PIN-PMN-PT crystal poled along the [011] direction. (**b**) Fitting curve of phase as a function of voltage. (**c**) Schematic of EO coefficient experimental device.

As-grown PIN-PMN-PT single crystals are optically opaque due to light scattering induced by their complex domain structures. To achieve transparency, we employed a high-temperature poling technique. This process suppresses light scattering by eliminating 109° domain walls while preserving 71° domains, thereby enhancing the electro-optic (EO) response. As shown in Figure 1a, the high-temperature-poled crystal displays a transmission of approximately 67% across the 0.4–2 μm wavelength range, approaching the theoretical limit when accounting for interface reflection. With anti-reflective coatings, the crystal achieves near-perfect transmission. The effective EO coefficient was measured using single-beam ellipsometry. As illustrated in Figure 1c, the experimental setup employed a He–Ne laser (633 nm) as the source, with light polarized at 45° via a Glan prism. With an applied electric field, the linearly polarized light undergoes phase retardation. The EO coefficient was derived from this phase shift using expression [16], by fitting the relationship between the phase shift and the electric field.

The resulting phase shift can be calculated by the following equation:

(2) $$\Delta \phi =\frac{\pi }{\lambda }n_{c}^{3}r_{c}\frac{V}{d}L$$

where λ is the wavelength of incident light; *n*_c_ is the refractive index of the sample; *γ_c_* is the effective electro-optic coefficient. The remaining parameters V, d and L are the strength of the applied electric field, gap between the electrodes, and transmission distance of the light wave in the sample, respectively.

The [011]-poled PIN-PMN-PT crystal exhibits a large effective electro-optic (EO) coefficient (*γ_c_*) of 452 pm/V, as determined from the linear fit to experimental data in Figure 1b. This value significantly exceeds the *γ_c_* of conventional LiNbO_3_ (20 pm/V). Density functional theory (DFT) calculations indicate that the enhanced EO response arises from combined ionic and piezoelectric contributions [32,33]. Unlike room-temperature poling, where irreversible electric-field-induced phase transitions (over-poling) degrade EO performance, high-temperature poling mitigates these effects by applying a reduced electric field, thereby preserving a high EO coefficient.

### 3.2. Nickel In-Diffusion Process

**Figure 2:**
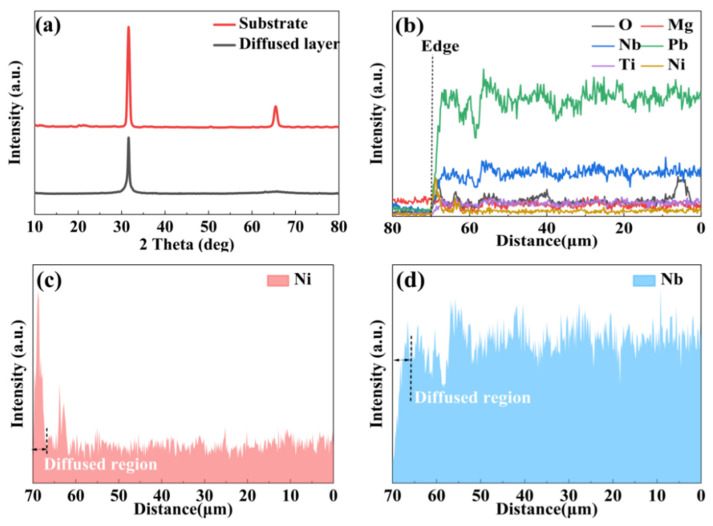
(**a**) X-ray diffraction patterns of PIN-PMN-PT crystal. (**b**) EDS spectra of elements in the PIN-PMN-PT crystal. (**c**,**d**) EDS scanning curves for the element of Ni and Nb, respectively.

The development of high-quality optical waveguides is critical for realizing PIN-PMN-PT-based integrated electro-optic modulators. In this study, we fabricated an optical waveguide in a rhombohedral PIN-PMN-PT single crystal via nickel in-diffusion. The impact of nickel diffusion on the crystal structure was analyzed using X-ray diffraction (XRD, D/Max-IIIC, Rigaku, Japan), while energy-dispersive X-ray spectroscopy (EDS) was employed to characterize the elemental distribution and diffusion depth. Comparisons between the diffused and un-diffused regions are presented in Figure 2.

Figure 2a presents the XRD patterns of the crystal before and after nickel diffusion. The pristine crystal exhibits a diffraction pattern characteristic of the rhombohedral phase. Following diffusion, the decreased intensities of the (220) and (110) peaks indicate that the primary crystal structure remains intact, although a fraction of the nickel atoms incorporates into the lattice via a solid-state reaction. Any residual surface nickel oxide was removed via polishing prior to subsequent device fabrication.

XRD patterns of the crystal before and after nickel diffusion are shown in Figure 2a. The un-diffused crystal exhibits characteristic rhombohedral phase diffraction patterns. After diffusion, the intensities of the (220) and (110) peaks decrease, indicating that the primary crystal structure is preserved while a fraction of nickel atoms undergoes a solid-state reaction with the lattice. A small amount of residual nickel oxide on the crystal surface was removed by polishing prior to further device fabrication.

As shown in Figure 2c, elemental depth profiling via EDS line-scan analysis reveals a sharp increase in nickel concentration at a depth of approximately 2.2 μm, beyond which it stabilizes. The nickel concentration within the diffusion layer is significantly higher than in the substrate. Conversely, in Figure 2d, the niobium concentration in the diffused region is lower than in the un-diffused area, indicating a nickel-diffused layer extending to about 2.2 μm. These phase structure analyses demonstrate that the nickel-diffused waveguide retains the original crystal phase and exhibits a significant electro-optic (EO) response. The effective EO coefficient of the [011]-poled rhombohedral crystal reaches approximately 452 pm/V, underscoring the potential of the nickel-diffused region for high-efficiency modulation.

**Figure 3:**
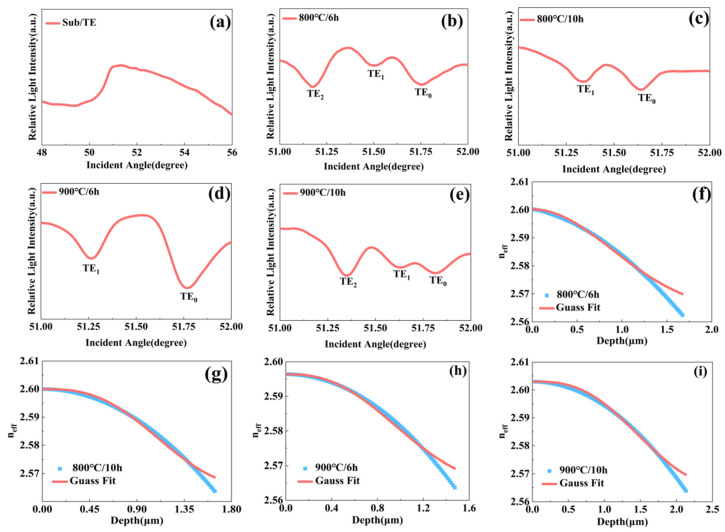
Patterns of relative light intensity of TE-polarized light at 633 nm as a function of the incident angle inside prism for: the substrate layer of the crystal (**a**), crystals coated with a 50 nm nickel film under the conditions of (**b**) 800 °C/6 h, (**c**) 800 °C/10 h, (**d**) 900 °C/6 h, and (**e**) 900 °C/10 h. (**f**–**i**): Gaussian fitting results of the corresponding experimental data from (**b**–**e**).

The prism coupling instrument is employed to characterize the refractive index change between the nickel-diffused region and the substrate crystal. The prism coupling instrument is effective in characterizing the refractive index, thickness, and guided wave modes of a thin waveguide layer [34]. As presented in Figure 3, the refractive indices of the waveguide modes were determined from the relative intensity profiles as a function of the incident angle. For the un-diffused crystal at an operating wavelength of 633 nm, TE polarization profile exhibited a single prominent dip, corresponding to a refractive index of 2.563 in Figure 3a. The waveguide properties strongly depend on diffusion conditions—specifically, temperature, duration, and nickel film thickness. As shown in Figure 3b–e, under different processing parameters, multiple guided modes were excited, confirming the formation of a planar waveguide structure. Each dip in the intensity profile corresponds to the effective refractive index of a guided mode, indicating that nickel in-diffusion successfully increased the refractive index to form a functional optical waveguide. Notably, the number and effective indices of the guided modes varied with diffusion conditions. Increasing the diffusion duration reduced the number of excited modes and lowered their effective refractive indices at 800 °C. In contrast, both the number of modes and the corresponding indices increased with longer diffusion times at 900 °C. This indicates that at 800 °C, excessive diffusion diminishes the refractive index enhancement, whereas at 900 °C, longer times are necessary to achieve sufficient nickel in-diffusion and the desired index increase.

The refractive index profile of the waveguide region was reconstructed using the Inverse Wentzel–Kramers–Brillouin (IWKB) method [35]. Based on the refractive index profiles under various conditions in Figure 3b–e, the resulting profiles could be well fitted with a Gaussian function in Figure 3f–i, from which key waveguide parameters were derived. The Gaussian function used here can be described by the following equation:

(3) $$n(x)=n_{s}+\Delta n\exp[-{\left(x/d\right)}^{2}]$$

where *n_s_* (index of the substrate), Δ*n* (refractive index change between the diffusion surface and the substrate), d (effective depth of the waveguide), d (effective depth of the waveguide), which equals to the depth at which the index change Δ*n* decreases to Δ*n*/*e*.

The refractive index profiles of the nickel-diffused waveguides, reconstructed using the inverse IWKB method, are presented in Figure 3f–i. The scatter points represent the reconstructed index data, while the red curves show the corresponding Gaussian fits. Key parameters derived from the Gaussian fitting are summarized in Table 1. For instance, at an operating wavelength of 633 nm, the surface index (n_s_), index change (Δ*n*), and diffusion depth (d) are 2.603, 0.04, and 1.6998 μm, respectively. The excellent agreement between the fitted parameters and the experimental data from the prism coupling method confirms that the nickel-diffused PIN-PMN-PT waveguide exhibits a graded-index profile well approximated by a Gaussian function.

**Figure 4:**
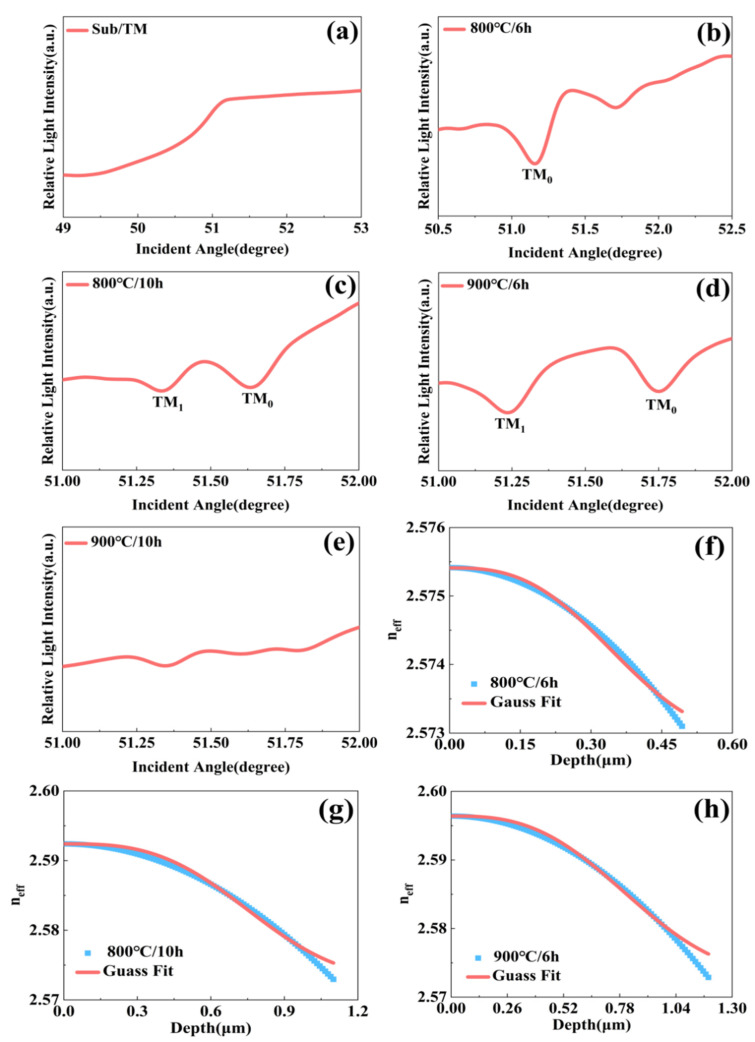
Patterns of relative light intensity of TM-polarized light at 633 nm as a function of the incident angle inside prism for: the substrate layer of the crystal (**a**), crystals coated with a 50 nm nickel film under the conditions of (**b**) 800 °C/6 h, (**c**) 800 °C/10 h, (**d**) 900 °C/6 h, and (**e**) 900 °C/10 h. (**f**–**h**): Gaussian fitting results of the corresponding experimental data from (**b**–**d**).

Waveguide characterization under TM polarization at 633 nm reveals distinct mode excitation behavior compared to TE polarization. As shown in Figure 4a–h, while multiple TM modes are supported in the nickel-diffused PIN-PMN-PT crystal, their excitation is generally less pronounced than for TE modes. A single distinct TM mode is observed after diffusion at 800 °C for 6 h. Extending the duration to 10 h at 800 °C increases the number of guided TM modes and enhances their effective refractive indices—a trend opposite to that observed for TE modes under identical conditions. After diffusion at 900 °C for 6 h, two TM modes are excited with higher effective indices. However, no TM modes are excited after 10 h at 900 °C.

Generally, the mode selectivity of this optical waveguide is determined by the anisotropic change in the crystal’s refractive index caused by nickel diffusion. Under specific process conditions, the crystal’s refractive index for ordinary light (*n_o_*) increases, while that for extraordinary light (*n_e_*) remains unchanged or even slightly decreases. This implies that for extraordinary light, the refractive index difference between the diffused region and the substrate crystal is minimal or even negative, preventing the formation of an effective waveguide structure. Consequently, the light cannot be confined and will rapidly radiate out. Therefore, the nickel diffused optical waveguide naturally supports only the propagation of ordinary light (corresponding to the TE mode), achieving mode selectivity.

The effective refractive index contrasts (Δ*n*) for the TM mode are 0.91% and 0.96%, lower than those for the TE mode, indicating weaker light confinement. Gaussian fitting of the index profile provides key waveguide parameters summarized in Table 2, confirming the capability of the nickel-diffused layer to transmit TM mode light at 633 nm. The clear predominance of TE mode excitation demonstrates that nickel-diffused PIN-PMN-PT waveguides are inherently more conducive to supporting TE modes.

Mode selectivity between TE and TM guided modes can be precisely controlled by optimizing diffusion time, temperature, and ambient atmosphere. This tunability is crucial for fabricating Y-branch waveguide devices in PIN-PMN-PT crystals for fiber optic gyroscope applications. The ferroelectric domain structure, which significantly influences crystal properties, was characterized using Piezoelectric Force Microscopy (PFM). Rhombohedral PIN-PMN-PT crystals typically exhibit a diffuse phase transition with a coexistence of micrometer-sized curved, strip-like domains and embedded nanodomains. Out-of-plane PFM amplitude images of crystals diffused at 800 °C, 850 °C, and 900 °C show consistent phase and amplitude responses, indicating minimal surface influence on PFM imaging. The domain morphology retains the characteristics of the rhombohedral phase. As diffusion temperature increases from 800 °C to 900 °C, the domain area enlarges, with only micrometer-sized domains observable at 900 °C. This reduction in domain wall density decreases light scattering, significantly enhancing crystal transparency.

**Figure 5:**
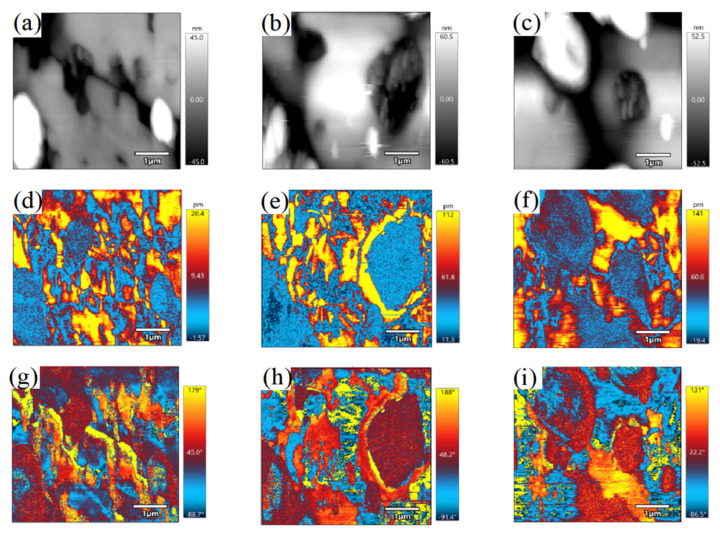
Diffusion temperature (800 °C, 850 °C, 900 °C) dependence of surface morphology (**a**–**c**), out-of-plane piezo-response (**d**–**f**), and phase images (**g**–**i**) in nickel-diffused PIN-PMN-PT single crystals, characterized by PFM.

The functional properties of relaxor ferroelectric crystals are strongly influenced by their ferroelectric domain configurations. Thus, understanding how nickel diffusion alters the domain structure is essential. In PIN-PMN-PT crystals, the domain architecture is intrinsically linked to the phase structure. Rhombohedral PIN-PMN-PT, owing to its relaxor nature, does not typically exhibit clearly resolvable macroscopic domains. Instead, its domain structure consists of a mixture of curved, strip-like domains spanning several square micrometers and embedded nanodomains, which collectively give rise to a diffuse phase transition. Here, we employed Piezoelectric Force Microscopy (PFM) to characterize the domain structures of PIN-PMN-PT crystals following nickel diffusion at various temperatures. Out-of-plane PFM amplitude images were successfully obtained for crystal diffused at 800 °C, 850 °C, and 900 °C for the first time. As illustrated in Figure 5, the PFM amplitude and phase images of the diffused regions are highly consistent at each temperature, indicating that the crystal surface state has a negligible impact on PFM imaging. Further analysis of phase and piezoelectric response maps confirms that the crystals retain distinct polarization responses, reflecting the typical domain morphology of rhombohedral PIN-PMN-PT. The crystal diffused at 800 °C exhibits irregular, sub-micrometer-scale domain patterns characteristic of the rhombohedral phase. With increasing diffusion temperature, as seen in Figure 5d–i, the domain area enlarges. In the sample treated at 900 °C, only micrometer-sized ferroelectric domains remain observable, while the nanodomains are largely undetectable. This coarsening of the domain structure reduces the density of light-scattering domain walls, resulting in a significant enhancement of optical transparency.

### 3.3. Simulation of Light Transmission

**Figure 6:**
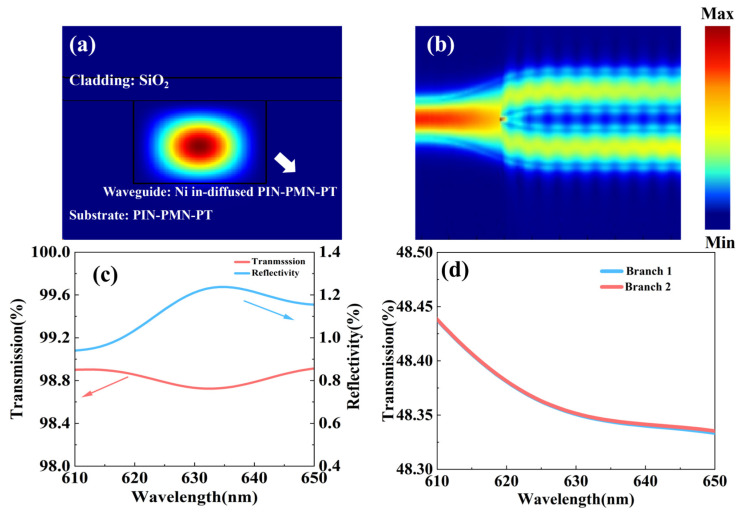
Simulated optical field of the cross section (**a**) and Y-branch (**b**); Transmission characteristic (**c**,**d**) of the PIN-PMN-PT Y-branch waveguide.

In order to investigate the optical transmission characteristics of the nickel-diffused PIN-PMN-PT waveguide, finite-difference time-domain (FDTD) simulations were performed. The model, shown in Figure 6a, consisted of a nickel-diffused PIN-PMN-PT core and a 1 μm thick silica buffer layer. The core, which served as the light-guiding region, had a width of 3.5 μm and a thickness of 1.6998 μm. We simulated the optical field distribution for TE polarization at a wavelength of 633 nm, with a substrate refractive index of 2.563 and a measured waveguide effective index of 2.603. Figure 6b illustrates a Y-branch structure based on this waveguide, designed with a 1000 μm transition length and a 200 μm waveguide separation. This design maintains a branch angle below 3°, which minimizing bending loss and suppressing mode conversion in the tapered section. The simulations confirm strong optical confinement within the waveguide and show that the Y-branch uniformly splits the input light into the two output arms, ensuring efficient transmission. Additionally, we simulated transmission and reflectivity of the waveguide. As shown in Figure 6c,d, the straight waveguide exhibits a transmission of nearly 99% across the 610–650 nm wavelength range. The two output arms of the Y-branch show equal transmission of, approximately 48.4%, the confirming equal-ratio beam splitting capability of the structure. Collectively, these results demonstrate the high-quality optical transmission enabled by the nickel-diffused PIN-PMN-PT waveguide, a key characteristic for the development of Y-branch modulators in fiber-optic gyroscopes.

## 4. Conclusions

In summary, the nickel in-diffusion method facilitates the fabrication of mode-selective optical waveguides within PIN-PMN-PT single crystals. Structural characterization confirms that both the crystalline integrity and the exceptional electro-optic (EO) response of the single crystal are preserved after nickel in-diffusion—a critical factor for high-performance modulation. The observed refractive index increase of up to 1.53% ensures robust optical confinement. Notably, under specific processing conditions, the waveguide exhibits exclusive TE mode excitation. This rigorous mode selectivity, combined with the giant EO response of the nickel-diffused PIN-PMN-PT platform, enables the realization of ultra-compact EO modulators with significantly reduced driving voltages. Such advancements are pivotal for the miniaturization and functional integration of next-generation fiber-optic gyroscopes.

## Figures and Tables

**Table 1:** Mode index and critical parameters derived from Gaussian fitting results of nickel-diffused PIN-PMN-PT crystals under various processing parameters (TE mode).

*Sample*	*T*/°C	*t*/h	*n_eff_*	Δ*n*/%	*D*/μm
1	800 °C	6 h	2.6002 (*x* = 0) TE_0_: 2.5958 TE_1_: 2.5872 TE_2_: 2.5757	1.42	1.2338
2	800 °C	10 h	2.6000 (*x* = 0) TE_0_: 2.5922 TE_1_: 2.5811	1.41	1.2258
3	900 °C	6 h	2.5964 (*x* = 0) TE_0_: 2.5962 TE_1_: 2.5758	1.18	1.1764
4	900 °C	10 h	2.6030 (*x* = 0) TE_0_: 2.5988 TE_1_: 2.5919 TE_2_: 2.5818	1.53	1.6998

**Table 2:** Mode index and critical parameters derived from Gaussian fitting results of nickel-diffused PIN-PMN-PT crystals under various processing parameters (TM mode).

*Sample*	*T*/°C	*t*/h	*n_eff_*	Δ*n*/%	*d*/μm
1	800 °C	6 h	2.5755 (*x* = 0) TM_0_: 2.5754	0.1	0.4931
2	800 °C	10 h	2.5924 (*x* = 0) TM_0_: 2.5921 TM_1_: 2.5814	0.75	0.8777
3	900 °C	6 h	2.5964 (*x* = 0) TM_0_: 2.5962 TM_1_: 2.5780	0.91	0.9616
4	900 °C	10 h	-	-	-

## Data Availability

The original contributions presented in this study are included in the article. Further inquiries can be directed to the corresponding authors.
